# Prehospital initiation of extracorporeal life support for refractory out-of-hospital cardiac arrest–results of a prospective observational study

**DOI:** 10.1186/s13054-026-05958-2

**Published:** 2026-03-19

**Authors:** Dirk Lunz, Alois Philipp, Walter Petermichl, Bernhard Graf, Matthias Lubnow, Christof Schmid, Maik Foltan, Markus Zimmermann, Peter-Paul Ellmauer, Bernhard Ulm, Sebastian Blecha, Thomas Müller

**Affiliations:** 1 Department of Anesthesiology, University Medical Center Regensburg, Franz-Josef-Strauß-Allee 11, Regensburg, 93053 Germany; 2 Department of Cardiothoracic Surgery, University Medical Center, Regensburg, Germany; 3 Department of Internal Medicine II, University Medical Center, Regensburg, Germany; 4 Emergency Department, University Medical Center, Regensburg, Germany; 5 University Medical Center Rechts der Isar, Munich, Germany

**Keywords:** Cardiopulmonary resuscitation, Extracorporeal circulation, Extracorporeal life support, Out-of-hospital cardiac arrest

## Abstract

**Background:**

Despite advances in cardiopulmonary resuscitation (CPR), survival after out-of-hospital cardiac arrest (OHCA) remains low. Use of veno-arterial extracorporeal membrane oxygenation (VA ECMO) as extracorporeal CPR (ECPR) may improve outcomes in refractory OHCA. We evaluated the effect on hospital discharge rate and neurological function of integrating on-scene ECPR into routine emergency care for refractory OHCA. Besides that we assessed predictors of unfavorable outcomes.

**Methods:**

A prospective observational study was conducted from October 2013 to September 2023 in Regensburg, Germany. A dedicated ECMO team was alerted 24/7 in parallel with standard emergency medical services for suspected OHCA. On-scene VA ECMO was initiated based on predefined inclusion/exclusion criteria. Patients were transported to a university medical center for guideline-based post-resuscitation care. Clinical data, including CPR parameters, initial physiology, and outcomes, were recorded and analyzed.

**Results:**

Over ten years, 11,235 alerts resulted in 2,655 (23.6%) on-scene evaluations of OHCA. VA ECMO was initiated in 213 patients with refractory OHCA (8.0% of on-scene CPR evaluations). The median time between beginning of CPR and start of VA ECMO was 45 min (IQR: 35–63). Median ECMO duration was 2 days (IQR 1–4). Survival to hospital discharge was 34.7% (74/213), with 89.2% (66/74) achieving a good neurological outcome and an independent daily living. In multivariable analysis restricted to on-scene variables, independently associated with unfavorable outcomes were: bilaterally dilated pupils (OR 5.79 [1.85–19.8]; *p* = 0.003), absence of bystander CPR (OR 4.38 [1.23–18.2]; *p* = 0.029), use of mechanical CPR devices (OR 5.53 [2.09–15.9]; *p* < 0.001), initial asystole (OR 35.0 [5.24–731]; *p* = 0.002), and CPR-to-ECMO interval > 45 min (OR 3.07 [1.09–9.14]; *p* = 0.037).

**Conclusions:**

Prehospital ECPR is feasible and can be integrated into a regional emergency medical system when performed by a highly experienced team. Survival rates in this selected cohort exceeded typical OHCA outcomes, with a high proportion of patients achieving favorable neurological recovery. Early VA ECMO initiation and several on-scene factors are key determinants of prognosis.

**Trial registration:**

German Clinical Trials Register. (DRKS00035400; URL: https://www.drks.de/search/de/trial/DRKS00035400)

**Graphical abstract:**

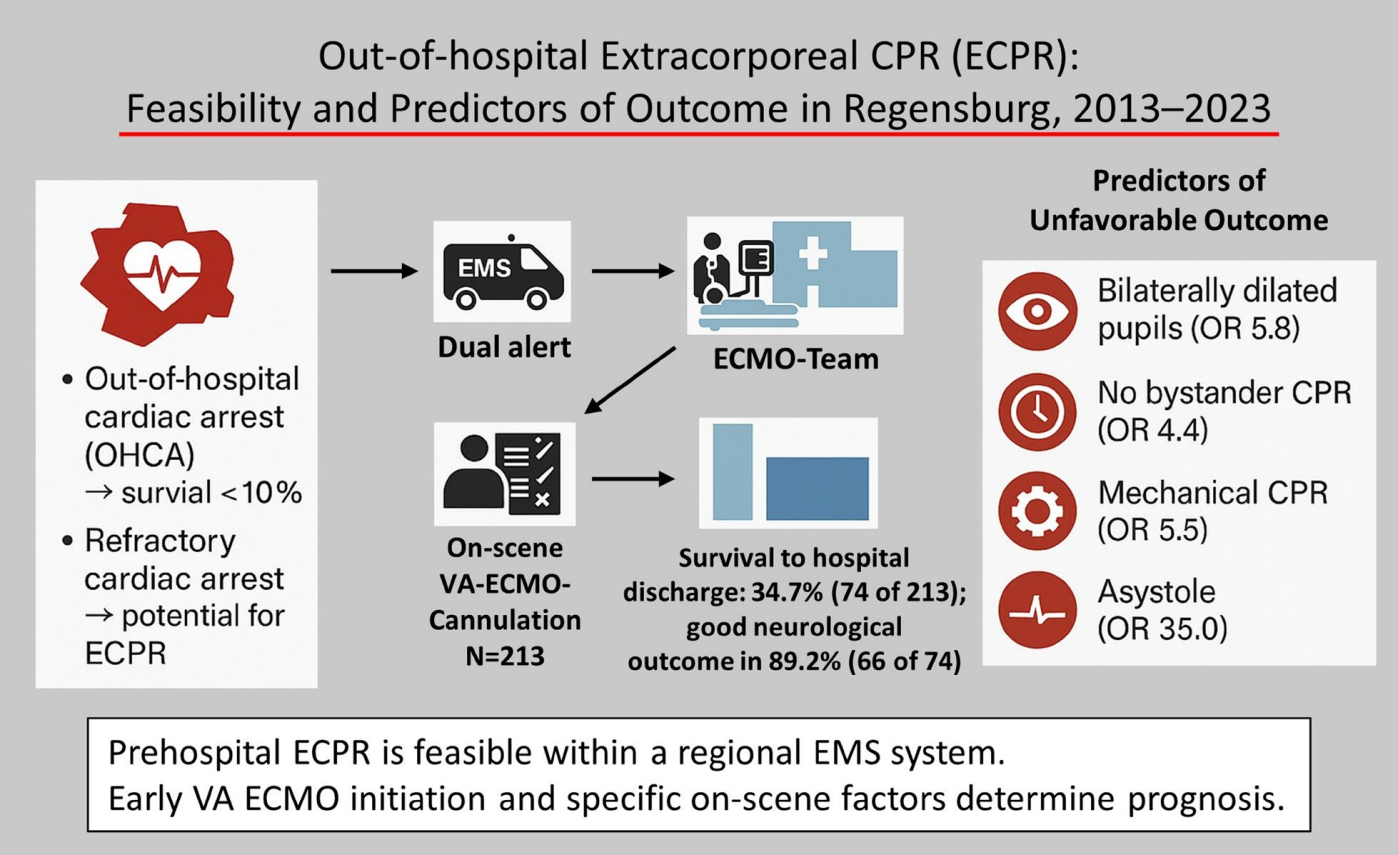

**Supplementary Information:**

The online version contains supplementary material available at 10.1186/s13054-026-05958-2.

## Background

Despite on-going medical advancements, sudden cardiac death due to out-of-hospital cardiac arrest (OHCA) remains one of the greatest challenges in modern emergency medicine. The incidence of approximately 62–100 cases per 100,000 population per year, combined with survival-to-discharge rates ranging from 2% in Asia to 11% in Australia and 9% in Europe, highlight the urgent need for continued research to improve dismal outcome rates [1–3].

Patients who fail to achieve return of spontaneous circulation (ROSC) despite conventional cardiopulmonary resuscitation (CPR) for an extended period—thus suffering from refractory OHCA—have a particularly poor prognosis. If CPR lasts longer than 15–20 min, the probability of a functionally favorable neurological outcome drops to approximately 2% [4]. Consequently, the scientific discussion in this field not only focuses on refining and further developing current CPR guidelines but also explores alternative treatment strategies.

Among these alternatives, mechanical CPR devices and extracorporeal life support systems (ECLS) have gained increasing attention. Recently, the effectiveness of currently available mechanical CPR devices in improving overall survival and neurological outcomes has been questioned, leaving ECPR as one of the promising future treatment options for CPR [5–7]. Over the past two years, several randomized controlled trials comparing conventional and extracorporeal cardiopulmonary resuscitation for OHCA have been published. While some studies, such as the INCEPTION Trial in the Netherlands, found no prognostic benefit of extracorporeal CPR (ECPR) [8], others reported improved survival in highly selected patients (ARREST Trial [9]) or better 180-day survival in patients without prehospital ROSC (Prague OHCA study [10]).

Available studies emphasize two crucial factors: patient selection and the necessity of a well-established infrastructure with an experienced team. Research from North America suggests that the optimal window to switch from one strategy (conventional CPR) to another (ECPR) balancing the risks and benefits of this invasive procedure—ranges from 8 to 24 min [11, 12]. Furthermore, fast initiation of ECPR minimizing low-flow time and shock manifestation, is widely recognized as essential, with CPR-to-ECMO times of less than 45 min potentially yielding the best neurological outcomes [13]. Meeting these prerequisites, combined with a stringent patient selection process based on strict criteria, may be the key to success.

Against this background, we conducted a prospective observational study over a 10-year period (RECA study: **R**egensburg **E**CLS for **C**ardiac **A**rrest) to evaluate whether a prehospital ECPR strategy for therapy-refractory OHCA can be integrated as a standard of care within the regular emergency system of a defined urban region in Germany. Additionally, we analyzed patient outcomes to identify simple, readily available prehospital risk factors for mortality. Based on our previously acquired scientific insights with ECPR [14], we hypothesized that prehospital initiation of ECPR for refractory OHCA would improve survival rates compared to historical outcomes by enabling faster return of spontaneous circulation (ROSC) and adequate organ perfusion.

## Methods

### Study design

This prospective observational single-center pilot study started at the University Hospital of Regensburg, Germany, in October 2013 and is still ongoing. Presented in this publication is the analysis of patient data for the first 10 years up to September 2023. The study was approved by the local institutional review board (Protocol no. 12-101-0266, approved in February 2013) and registered with the German Clinical Trials Register (DRKS00035400).

### Patient cohort and indication

Patient enrollment was performed in close collaboration with the Emergency Medical Dispatch Center (EMDC) of Regensburg, being responsible for the city and the county of Regensburg (covering more than 330,000 people in an area of about 1400 km^2^). Analogous to other prehospital ECPR programs like for example the one in Paris the ECPR team, located at the University Medical Center, was informed 24/7 in every case of a suspected or confirmed OHCA via pager parallel to the regular emergency service. By immediate telephone consultation, patients that suffered from an unwitnessed OHCA with unknown arrest-time, age over 75 years, patients with traumatic cardiac arrest, known preexisting multi-morbidity and patients with definite signs of death were excluded. In all other cases the ECPR team was dispatched and the OHCA situations were evaluated on scene. Indications for implementation of prehospital ECPR were an age between 18 and 75 years, a witnessed OHCA and no ROSC after 15 min of ACLS, a maximum of 10 min until start of layperson or professional CPR, recurrent need for CPR despite transient ROSC or definite signs of life. Neither the type of first recorded heart rhythm nor the type of heart rhythm at the time of on-scene evaluation were part of the indication setting. An overview of our checklist of (contra-) indications for prehospital VA ECMO implantation is showed in Supplement Table S1.

### ECPR Team

The ECPR Team responsible for reviewing the correct indication and performing the ECMO implementation on scene consisted of an ECMO specialist and a clinical perfusionist. The physician had to be a consultant of cardiac anesthesiology and intensive care with more than five years of clinical practice, proven expertise in emergency medicine and verified experience in providing care to ECMO patients.

### Patient care during refractory OHCA

After arrival on scene, the ECPR team reevaluated the patient’s actual status and confirmed the correct indication based on the underlying information. In case a decision towards ECPR was made, the regular emergency physician responsible for the patient also had to agree with the procedure. If possible approval of relatives was obtained. After performing a fast ultrasound of groin vessels in most cases, ECMO implementation in the femoral artery and vein was done by using a landmark-based Seldinger technique. For venous drainage we used 21–23 French, 55 cm long cannulas whereas arterial return was achieved by 15–17 French and 15–23 cm long cannulas. After placing the guidewire and the dilators for the first cannula, according to the patient’s medical history a bolus of 5000 I.U. heparin was administered. ECMO blood flow was stepwise increased up to 50 ml/kg ideal body weight/min with a sweep flow of about 2 l/min and a F_i_O_2_ of 50%. After successful implantation (5–7 min usually), all patients were transported to the University Medical Center of Regensburg. The subsequent diagnostic investigations (immediate whole body CT scan and coronary angiography in almost all cases) and the post resuscitation intensive care including targeted temperature management and a patient-specific multimodal neuro-prognostication were performed according to actual guidelines and institutional standards [15, 16].

### Registration of cardiac arrest and ECPR characteristics

Beside the VA ECMO parameters (days of support, lowest body temperature under running ECMO, time for cannulation, cannulation-related complications and technical ECMO problems), the following additional parameters were documented: witnessed OHCA, layperson CPR, distance to hospital, type of transportation (ground vs. air based) and type of resuscitation (manual vs. mechanical), initial cerebral regional hemoglobin oxygen saturation (INVOS), initial cardiac rhythm and pupil status, time between collapse and start of CPR by trained professionals, duration between start of CPR and beginning of ECMO circulation and duration of transportation with VA ECMO to the emergency department.

### Registration of laboratory and patient-related parameters

After ECPR and hospital admission, the following parameters were recorded: age, body mass index (BMI), initial hemoglobin and lactate value, maximum of neuron-specific enolase (NSE) and days of ECMO support, duration of ICU and hospital stay and survival rate. Functional neurological outcome of ECPR survivors were measured at hospital discharge using the Cerebral Performance Category (CPC) score, which categorizes patient outcomes into five categories (good recovery [CPC1], moderate disability [CPC2], severe disability [CPC3], permanent vegetative state [CPC4] and death [CPC5]) [17, 18]. Additionally, each survivor was contacted by phone call once a year after hospital discharge and interviewed to activities of daily living and possible health limitations. According to the interview, patients were classified to current CPC status.

### Study aims

The primary aim of this study was to analyze the hospital discharge rate and neurological function of ECPR survivors. Secondary aims were to analyze patient, laboratory and ECMO-treatment parameters before and after ECPR, which were associated with mortality during or after VA ECMO therapy until hospital discharge. Because preliminarily used data on patient characteristics, arrest and timing of CPR often had to be corrected later with more information becoming available, outcome were also analyzed according to these settings.

### Statistical analysis

All statistical analyses in this study were performed by an independent statistic office: Independent statistical consulting Bernhard Ulm, Munich.

### Sample size considerations

This study was designed as a pilot study with several primary variables to get insights into the feasibility of routinely integrating on scene ECPR implementation for therapy-refractory OHCA in a given emergency medical system and into the detection of risk factors for hospital mortality during ECMO treatment after OHCA. Thus, no a priori sample size calculation could be performed [19, 20].

### Statistical methods

Data are presented as mean (± SD) or median (IQR) for continuous variables, depending on the underlying distribution, and as absolute and relative frequencies for categorical variables. Based on the current study, a cut-off value of 70 µg/L for NSE was applied in both univariate and multivariate analyses [21]. A p-value < 0.05 was considered statistically significant. All analyses were conducted using R software (version 3.5.1; www.r-project.org**).**

## Results

**Fig. 1:**
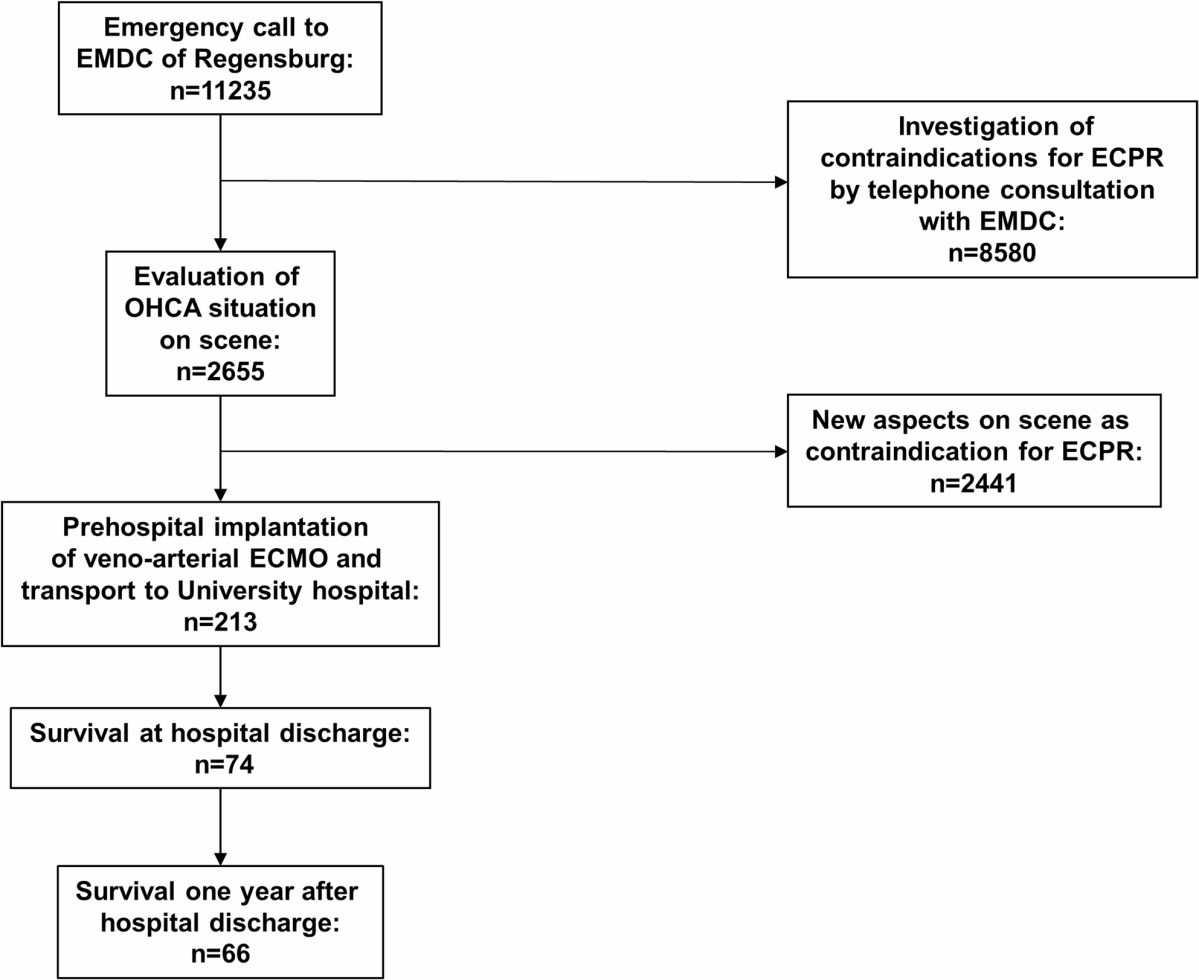
Flowchart of OHCA evaluation and ECPR indication over ten years. ECMO: extracorporeal membrane oxygenation; ECPR: extracorporeal cardiopulmonary resuscitation; EMDC: emergency medical dispatch center; OHCA: out-of-hospital cardiac arrest

In 11,235 cases, our ECPR team was notified by the local EMDC about a suspected or confirmed CPR situation. On-scene evaluation was conducted in 2,655 cases (23.6% of all emergency calls), and ECPR was implemented in 213 cases (8.0% of on-scene evaluations; see Fig. 1). 2442 patients already met exclusion criteria at the time of our arrival on scene.

### Characteristics and outcome of ECPR patients

The median age of all ECPR patients was 56 years (IQR: 49–65), the median body mass index was 26.3 kg/m^2^ (IQR: 24.2–29.4) and the majority of them were male (*n* = 163, 76.5%). 52.6% of patients were manually resuscitated, 47.4% with support by a mechanical CPR device. The time for ECMO implantation on scene was less than 10 min in 85.1% of the cases (181/213 cases). The median time between beginning of CPR and start of VA ECMO was 45 min (IQR: 35–63) and the median subsequent transport time was 45 min (IQR: 35–60). Acute cannulation-related complications (including cannulation failure in 4 patients) occurred in 37 patients, while ECMO associated technical problems were observed in 18 cases. These are listed in more detail in Supplement Tables S2 and S3. The causes for OHCA are listed in Table 1. The most common cause was coronary heart disease (CHD) with an acute coronary syndrome (ACS) in 57.3%, another 26.2% of patients suffered from primary heart rhythm disturbances without ACS.

**Table 1:** Cause of OHCA (*n* = 213)

Cause	*N* (%)
Acute coronary syndrome including acute myocardial infarction)	122 (57.3)
Ventricular fibrillation / tachycardia, no ACS	38 (17.8)
Pulseless electrical activity / asystole, no ACS	18 (8.4)
Others (pulmonary embolism, near drowning, sepsis, aortic dissection, cardiomyopathy, intoxication)	35 (16.5)

ACS: Acute coronary syndrome

Table 2 (see at the end of manuscript) demonstrates the characteristics of ECPR survivors and non-survivors. For this analysis, not initially used preliminary characteristics at time of cannulation, but verified information’s, which often became available only later, were used. Overall, 34.7% (74 of 213) of all ECPR patients survived to hospital discharge, with the vast majority of survivors presenting with a favorable neurological outcome (89.2% [66 of 74 patients] CPC 1 or 2). The main cause of hospital mortality was cerebral hypoxia in 70.5% of all deceased patients, followed by refractory low cardiac output in 10.8% (see Supplement Table S4). One year after hospital discharge, 31.0% (66 of 213 patients) were alive. Seven of eight survivors with CPC 3 and 4 at hospital discharge died during 12 months follow up.

**Table 2:** ECPR survivors vs. non-survivors (*n* = 213)

Variables	Survivors (*n* = 74)	Non-Survivors (*n* = 139)	*p*-value	Missing [*n*]
Demographic parameters				
**Age** (years) median (IQR)	57 (49–66)	56 (49–65)	0.6	-
**Sex** n (%) Female Male	15 (20.3) 59 (79.7)	35 (25.2) 104 (74.8)	0.42	-
**BMI** (kg × m^2^) median (IQR)	26.3 (24.1–29.4)	26.4 (24.2–29.4)	0.57	-
***CPR/ECPR characteristics***				
**Unwitnessed OHCA** n (%) Yes No	4 (5.4) 70 (94.6)	47 (33.8) 92 (66.2)	< 0.001*	-
**Bystander CPR within 10 min** n (%)	57 (77.0)	62 (44.6)	< 0.001*	28
**Type of CPR** n (%) Manual Mechanical	58 (78.4) 16 (21.6)	54 (38.8) 85 (61.2)	< 0.001*	-
**Time until professional CPR ≥ 15 min** n (%)	8 (10.8)	33 (23.7)	< 0.001*	39
**Initial pupil status** n (%) Bilaterally dilated Bilaterally small to regular	9 (12.2) 65 (87.8)	81 (58.3) 22 (41.7)	< 0.001*	-
**Initial cerebral INVOS** (%) median (IQR)	41 (32–50)	27 (18–42)	< 0.001*	80
**Initial cardiac rhythm** n (%) Ventricular fibrillation Pulseless electric activity Asystole	64 (86.5) 9 (12.2) 1 (1.4)	63 (45.3) 31 (22.3) 45 (32.4)	< 0.001*	1
**Time begin CPR to start ECMO** ≥ 45 min n (%)	22 (29.7)	82 (59.0)	< 0.001*	79
**Type of transport** n (%) Ground-based Air-based	54 (73.0) 20 (27.0)	114 (82.0) 25 (18.0)	0.12	-
**Transport distance to on-scene CPR** (km) median (IQR)	15 (8–30)	15 (8–29)	0.59	-
***Patient-related characteristics at and after hospital admission***				
**Minimum measured temperature at admission** (°C) median (IQR)	33.9 (32.8–34.2)	33.4 (32–34)	0.079	3
**Initial hemoglobin** (mg/dl) median (IQR)	11.4 (10.4–12.9)	10.3 (9.1–11.8)	< 0.001*	-
**Initial lactate** (mg/dl) median (IQR)	73 (52–104)	121 (95–152)	< 0.001*	-
**Peak NSE (ng/ml)** median (IQR)	44 (35–55)	191 (106–352)	< 0.001*	24
**ECMO support** (days) median (IQR)	3 (2–4)	2 (1–3)	< 0.001*	-
**ICU stay** (days) median (IQR)	15 (11–24)	3 (1–5)	< 0.001*	-
**Hospital stay** (days) median (IQR)	22 (17–31)	3 (1–5)	< 0.001*	-

*p < 0.05 in bold; entries depict the median (IQR) or frequencies in percentage; BMI: body mass index; CPR: cardiopulmonary resuscitation; ECMO: extracorporeal membrane oxygenation; ICU: intensive care unit; INVOS: in-vivo optical spectroscopy, cerebral regional tissue oxygen saturation; NSE: neuron-specific enolase; OHCA: out-of-hospital cardiac arrest

### Concordance of preliminary inclusion criteria at cannulation and later available verified information

**Fig. 2:**
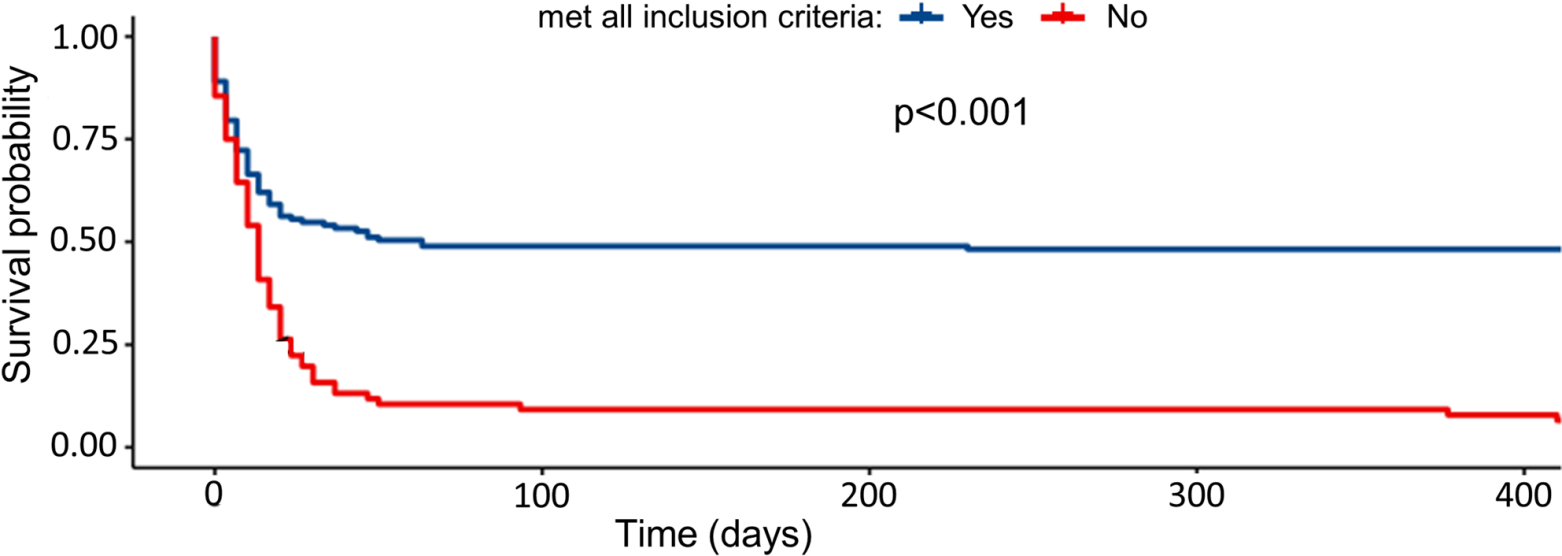
Accordance of inclusion criteria of ECPR patients on survival rate

Following repeated reassessment and rigorous validation over several days post-admission, only 64.3% (137/213) of the initially eligible ECPR patients—who had met the inclusion criteria at the scene with preliminary information—continued to fulfill the predefined eligibility criteria for ECPR: age 18–75 years, observed arrest, layperson CPR within 10 min and lack of preexisting multi-morbidity (see Table S1). The criterion “layperson CPR within 10 minutes” was not met in 27 out of 76 patients (35.5%), while “observed arrest” was not fulfilled in 42 out of 76 patients (55.3%). Both criteria being incorrect simultaneously occurred in 7 out of 76 patients (9.2%). Figure 2 presents the survival outcomes stratified by fulfillment of all inclusion criteria before cannulation and after retrospective verification. Of 137 patients truly fulfilling the inclusion criteria 67 (48.9%) survived to discharge from hospital. In multivariate analysis, OHCA patients who in fact satisfied all prehospital inclusion criteria for VA ECMO exhibited a more than fourfold increase in the odds of hospital survival rate (OR: 4.34 [1.5–14.0], *p* = 0.009).

### Risk factors for mortality after prehospital ECPR in OHCA patients

The results of the univariate analysis on ECPR characteristics are demonstrated in Table 3.

**Table 3:** Univariate analysis of ECPR characteristics (*n* = 213)

Variables	Odds Ratio (95%-CI)	*p*-value
Age	1.00 (0.99–1.00)	0.62
Sex	1.06 (0.91–1.24)	0.42
BMI	1.00 (0.99–1.01)	0.57
Unwitnessed OHCA	1.41 (1.22–1.63)	< 0.001*
No Bystander CPR within 10 min	1.50 (1.31–1.71)	< 0.001*
Mechanical CPR	1.43 (1.27–1.61)	< 0.001*
Time until professional CPR ≥ 15 min	1.32 (1.13–1.55)	< 0.001*
Initial pupil status: Bilaterally dilated	1.53 (1.37–1.72)	< 0.001*
Initial INVOS ≤ 18	1.56 (1.26–1.94)	< 0.001*
Initial cardiac rhythm Pulseless electrical activity Asystole	1.32 (1.13–1.54) 1.62 (1.40–1.88)	< 0.001* < 0.001*
Time CPR-to-ECMO > 45 min	1.30 (1.15–1.48)	< 0.001*
Type of transport	1.13 (0.97–1.32)	0.12
Transport distance	1.00 (0.99–1.02)	0.59
Minimum measured temperature at admission	0.97 (0.94–1.01)	0.079
Hemoglobin at hospital admission	0.94 (0.91–0.97)	< 0.001*
Lactate at hospital admission	1.01 (1.00–1.01)	< 0.001*
Peak NSE > 70 µg/L after hospital admission	1.93 (1.75–2.13)	< 0.001*

*p < 0.05: in bold; BMI: body mass index; CPR: cardiopulmonary resuscitation; ECMO: extracorporeal membrane oxygenation; ECPR: extracorporeal cardiopulmonary resuscitation; INVOS: cerebral regional hemoglobin oxygen saturation; NSE: neuron-specific enolase; OHCA: out-of-hospital cardiac arrest

The results of the comprehensive multivariable analysis, encompassing all (pre- and in-hospital) collected parameters, are summarized in Table 4. In this analysis, the absence of immediate bystander CPR, an initial presentation with asystole, and a NSE level greater than 70 µg/L were independently associated with unfavorable outcomes.

**Table 4:** Multivariable logistic regression models on all ECPR characteristics

Independent variable	Dependent variable	Adjusted		p-value
		OR	95% CI	
Hospital death	Age	1.05	(0.98–1.13)	0.18
	Sex	0.46	(0.04–4.55)	0.51
	BMI	0.95	(0.84–1.06)	0.38
	Unwitnessed OHCA	0.99	(0.08–15.4)	> 0.99
	No Bystander CPR within 10 min	14.7	(1.99–175)	0.016*
	Mechanical CPR	2.97	(0.59–16.0)	0.19
	Time until professional CPR ≥ 15 min	3.85	(0.48–33.0)	0.20
	Initial pupil status: Bilaterally dilated	3.17	(0.54–21.6)	0.21
	Initial cardiac rhythm: Pulseless electrical activity Asystole	2.13 43.7	(0.28–19.0) (2.46–2.50)	0.47 0.028*
	Time CPR-to-ECMO > 45 min	4.79	(0.93–30.2)	0.072
	Type of transport	5.37	(0.59–62.1)	0.15
	Transport distance	1.01	(0.95–1.09)	0.67
	Minimum measured temperature	1.28	(0.89–2.01)	0.19
	Hemoglobin at hospital admission	0.66	(0.41–1.01)	0.054
	Lactate at hospital admission	1.00	(0.97–1.02)	0.71
	Peak NSE > 70 µg/L after hospital admission	46.5	(9.24–384)	< 0.001*

*p < 0.05: in bold; CPR: cardiopulmonary resuscitation; ECMO: extracorporeal membrane oxygenation; OHCA: out-of-hospital cardiac arrest

In the adjusted multivariable analysis limited to on-scene available ECPR-related variables, the following factors were independently associated with unfavorable outcomes: bilaterally dilated pupils as the initial pupillary status, absence of immediate bystander CPR, use of mechanical CPR devices, an initial non-shockable rhythm, and a time interval exceeding 45 min between the onset of CPR and the initiation of VA ECMO support (see Table 5).

**Table 5:** Multivariable adjusted logistic regression models of on-scene ECPR characteristics

Independent variable	Dependent variable	Adjusted		p-value
		OR	95% CI	
Hospital death	Unwitnessed OHCA	3.63	(0.74–20.3)	0.12
	No Bystander CPR within 10 min	4.38	(1.23–18.2)	0.029*
	Mechanical CPR	5.53	(2.09–15.9)	<0.001*
	Time until professional CPR ≥ 15 min	1.15	(0.29–4.56)	0.84
	Initial pupil status: Bilaterally dilated	5.79	(1.85–19.8)	0.003*
	Initial cardiac rhythm: Pulseless electrical activity Asystole	4.44 35.0	(1.31–17.14) (5.24–731)	0.021* 0.002*
	Time CPR-to-ECMO > 45 min	3.07	(1.09–9.14)	0.037*

*p < 0.05: in bold; CPR: cardiopulmonary resuscitation; ECMO: extracorporeal membrane oxygenation; OHCA: out-of-hospital cardiac arrest

## Discussion

This study describes the experience and results of a prehospital ECPR program firmly implemented into a regional regular emergency system in Germany over a 10-year period. The main findings were: in selected patients who suffered from refractory OHCA and were treated with prehospital VA ECMO, the overall survival rate was 34.7%. The majority of survivors presented with a favorable neurological outcome (89.2%).

Secondary findings were: (1) initially bilateral dilated pupils, absence of immediate bystander CPR, resuscitation with mechanical CPR devices, an initial non-shockable rhythm (especially asystole), longer duration between the beginning of CPR and the start of VA ECMO, were associated with a higher risk of hospital mortality after OHCA with ECPR. (2) OHCA patients who truly fulfilled all predefined inclusion criteria for prehospital VA ECMO (verified during follow-up) had a significantly better survival rate.

Several aspects in the difficult field of ECPR for OHCA deserve particular attention. Development and ongoing optimization of the essential infrastructure to deploy an effective ECPR service required more than two years to organize different topics like implementing parallel alerts in any suspected or proven resuscitation situation, 24/7 availability of an experienced team and transfer options (dedicated ECMO car or helicopter) to reach the scene of arrest as fast as possible. In addition to the necessary infrastructure, careful selection of patients, time to ECMO and subsequent dedicated intensive care treatment after arrival to hospital are important prerequisites for success. Besides that these data emphasize that there is a noteworthy difference in outcome between non-shockable rhythms: In PEA patients, 9/40 (23%) survived, whereas in asystolic patients only 1/46 (2%).

### Outcome of ECPR patients

OHCA with professional conventional CPR affected approximately 60.000 patients in Germany in 2022 with a hospital discharge rate of 11% and good neurological outcome (CPC 1–2) of 7% [22]. Registry based data from 28 countries in Europe described comparable results with 8% of OHCA patients and initial ROSC being discharged from the hospital alive [23].

In our study, we demonstrated a survival to hospital discharge of 34.7%, with nearly 90% of ECPR survivors achieving a good neurological outcome. One year after hospital discharge, we observed a survival rate of 31%. Besides the crucial role of bystander-performed CPR, which has been shown in previous studies, the essential factor for improving survival in patients with refractory cardiac arrest seems to be the rapid restoration of adequate blood circulation facilitated by VA ECMO. In a retrospective observational study of prehospital ECPR patients in Paris with a low-flow time of 75 min median a survival rate of 21% with good neurological outcome after one year was reported [24]. An Australian study reported a survival rate of 27% in 11 OHCA patients supported by ECPR [25]. Similarly, a large retrospective multicentric study in Japan analyzing 1,644 OHCA patients treated with ECPR found a survival rate at hospital discharge of 27.2%, though the proportion with favorable neurological outcome was significantly lower at 14.1% [26]. A systematic review of nine studies reported that ECPR in OHCA patients resulted in good neurological outcomes in 15–20% of cases, suggesting that the duration of cardiac arrest is a more critical factor than its location [27]. The latest international report from the Extracorporeal Life Support Organization (ELSO) registry indicated a survival-to-hospital discharge rate of 29.5% for ECPR patients in general. However, it has to be emphasized that in this report both, patients with in-hospital and out-of-hospital cardiac arrest were included; besides no information about the neurological status was given [28].

### Risk factors for mortality after pre-hospital ECLS in OHCA

Several factors, which can potentially be modified, seem to influence mortality when providing ECPR in OHCA. Time to ECMO appears to be critical, as has repeatedly been shown. Many studies report “CPR to ECMO time”, because in contrast to collapse, for CPR start often the precise time point can be elicited. Duration of CPR before ECPR initiation was a decisive factor for patient outcomes in the current study. Patients who were conventionally resuscitated for more than 45 min before the start of VA ECMO had a threefold increased risk of death. Several previous studies reported a clear correlation between low-flow duration and patient outcome [26, 29–31]. The randomized controlled Inception trial documented an arrest to ECMO time of 74 (63 to 87) minutes; ECMO was started in 52 patients out of 70 allocated to the ECPR arm, of whom 5 were discharged from hospital [8]. A secondary analysis of the Prague OHCA trial reports a good neurological outcome with CPC 1 and 2 after 180 days in 21.7% of 92 patients with no ROSC, who received ECPR [10]. Time to ECMO was 61 (55 to 70) minutes [32]. As noted, time to ECMO in the current study was 45 (35 to 63) minutes with a good neurological outcome in 31% of patients at hospital discharge. The low-flow time in our study was therefore comparable to the results of the CHEER-3 trial in Melbourne, in which—albeit in only a small number of patients—a hospital discharge rate of 40% was ultimately observed [33]. This underscores the crucial role of time; highlighting that achieving ECMO implementation within 45 min may be key. Transport of a patient with ongoing resuscitation not only is time consuming, but also may independently increase the risk of death [34]. Therefore, avoiding transport under CPR and instead transporting the ECMO team to the scene of arrest can save valuable time, which may be important. This approach has been successfully implemented for the first time in this pilot study. Timing can further be improved, if a very experienced team shortens time of cannulation. In this study on average, it took the ECPR team less than 10 min to implement ECMO on scene. The Inception trial described a median duration for cannulation of 20 (11 to 25) minutes [8]. The here reported results strongly support the assumption, that a prehospital instead of an emergency department implementation of ECPR can significantly shorten low-flow times in refractory cardiac arrest. However, it has to be stressed that to save time every single step of the rescue chain has to be optimized, which encompasses infrastructural, organizational and educational aspects. One example, which definitely reduced time to ECMO, was the parallel alert of the ECMO team with the general emergency team by the EMDC.

It is well conceivable that providing a highly invasive procedure in a foreign environment under suboptimal conditions may increase the rate of serious complications. In the current study cannulation-related complications were observed in 17.4% and technical ECMO problems in 8.5% (Table S2). These numbers are comparable to previously published data: a large retrospective study from Japan reported cannulation and ECMO‑related complications in 21.2% and 3.1%, respectively [26], even though OHCA patients were predominantly cannulated in the emergency department or in the catheterization lab. In this context, point-of-care-ultrasound (POCUS) during cannulation has become standard of care offering a periprocedural safety comparable to that of fluoroscopy [35, 36]. In January 2021, the routine use of ultrasound was implemented for all cannulations in the RECA study. In consequence, cannulation-associated complications were reduced from 21.9% to 9.2% (Table S3). Based on this experience, we strongly recommend the use of ultrasound during cannulation for ECPR also pre-hospital.

Bystander CPR also seems to be a decisive parameter in any prehospital ECPR program. Compared with no bystander CPR, a recent study of a US observational registry of OHCA patients found that bystander CPR, when initiated within 10 min, was associated with improved survival [37]. In our multivariable analyses, we also demonstrated about fourfold better survival following bystander CPR within 10 min in OHCA patients treated with ECPR.

Rather unexpectedly, the use of mechanical CPR devices (Lucas^®^, Autopulse^®^ or Corpuls CPR^®^ - not specifically differentiated) in the current study was independently associated with increased in hospital mortality (OR 5.53, 95% CI 2.09–15.9, *p* < 0.001). The decision to use such a device was always made by the responsible emergency physician on scene and was never determined by the ECPR team. In most cases, the primary rationale was to free up personnel. Interestingly, and contrary to our expectations, we observed no correlation between CPR duration and the use of a mechanical CPR device. Although it is too early for a final assessment of this technique in the field of OHCA, our findings are worrying and in line with previous results [5–7]. Whether this may be a consequence of incorrect management, a lower efficacy of chest compression, a higher complication rate with injury of bystander organs or asynchrony with controlled ventilation remains unclear. A limitation that should be acknowledged is that the application of mCPR was not guided by a standardized protocol. Consequently, the possibility of operator-dependent selection bias cannot fully be excluded, as emergency physicians may have been more likely to initiate mCPR in patients perceived to have a poorer prognosis. Yet, in particular the combination of mechanical device CPR with ECPR may lead to a higher number of serious complications such as severe intrathoracic hemorrhage [38–40], and we would suggest to refrain from its use if possible. Nonetheless, attention must be paid to maintaining maximal compression quality until the initiation of ECPR.

Without dispute correct indication and careful evaluation of a patient’s candidacy for on-scene ECPR are of great importance for outcome [41, 42]. However, due to the uncertainties of the pre-hospital emergency environment and due to the need to minimize low flow duration, the robustness of preliminary information on scene often is limited. In the present study, we could demonstrate that survival was significantly better if all inclusion criteria were truly fulfilled. Only about two thirds (137/213) of the patients initially considered eligible for ECPR with preliminary information continued to fulfill the predefined inclusion criteria afterwards. Had ECPR only been performed in this cohort, survival to hospital discharge would have been at more than 60%.

### Strengths and Limitations

To our knowledge, this study presents the largest prospective cohort of patients with OHCA, in whom prehospital ECPR was conducted. Over a period of 10 years, it has been integrated into the regional emergency medical system. With the implementation of rigorous standards and optimization of the entire infrastructure of the rescue chain, the average time from CPR to ECMO was 45 min. Cannulation was conducted by few operators available 24/7 with a high degree of experience.

Limitations include the single-center, single-region design, which may limit generalizability. Both provision of out of hospital ECPR and treatment of patients after cannulation was executed in a very experienced ECMO center. Absence of a control group prevents definitive conclusions about efficacy of ECPR. On-scene inclusion assessment may have varied by provider, introducing a potential selection bias.

## Conclusion

Initiation of ECPR for refractory OHCA is feasible in carefully selected patients pre-hospital at the scene of arrest. It can be associated with a survival rate with good neurological outcome exceeding that of conventional treatment. Important predictors of mortality include a prolonged low-flow time (> 45 min), absence of bystander CPR, initial asystole, use of mechanical CPR devices, and bilaterally dilated pupils. Rapid response logistics and adherence to predefined inclusion criteria, though limited by the reliability of information on scene, are essential for optimizing outcomes. Our findings support the integration of pre-hospital ECPR into advanced resuscitation strategies for selected OHCA patients, if a highly experienced ECMO team is available.

## Supplementary Information

Below is the link to the electronic supplementary material.

Supplementary Material 1.

## Data Availability

No datasets were generated or analysed during the current study.

## Abbreviations

BMI: Body mass index
CHD: Coronary heart disease
CPC: Cerebral performance category
CPR: Cardiopulmonary resuscitation
EMDC: Emergency medical dispatch center
ECLS: Extracorporeal life support systems
ECPR: Extracorporeal cardiopulmonary resuscitation
ELSO: Extracorporeal Life Support Organization
ICU: Intensive care unit
NSE: Neuron-specific enolase
OHCA: Out-of-hospital cardiac arrest
POCU: Point-of-care-ultrasound
ROSC: Return of spontaneous circulation
VA ECMO: Veno-arterial extracorporeal membrane oxygenation
