# Supplementary material for: Prehospital initiation of extracorporeal life support for refractory out-of-hospital cardiac arrest–results of a prospective observational study
Source: Crit Care. 2026 Mar 19;30:163. doi: 10.1186/s13054-026-05958-2 (PMC13064238; doi:10.1186/s13054-026-05958-2)
Supplement: Supplementary file 1 — Supplementary Material 1. [file 13054_2026_5958_MOESM1_ESM.docx]

# Supplementary files:

**Table S1:** Checklist of (contra-) indications for pre-hospital ECPR

| **Indications** | **Contraindications** |
| --- | --- |
| - age between 18 to 75 years | - age over 75 or under 18 years |
| - witnessed OHCA | - unwitnessed OHCA (or unknown) |
| - no ROSC after 15min of ACLS | - traumatic cardiac arrest |
| - maximum of 10 min until start of laymen or professional CPR | - unknown laydown period or more than 10 min until start of laymen/professional CPR |
| - recent need for CPR despite transient ROSC | - known preexisting multi-morbidity |
| - “Signs of Life” during conventional CPR | - definite signs of death |

*Notes: In our study, indications and contraindications were designed to be concordant yet complementary. From a practical standpoint, contraindications were considered of greater importance than indications: the presence of any known contraindication precluded the performance of ECPR. Consequently, the absence of any known or confirmed contraindication was mandatory for proceeding with ECPR.*

*ACLS: advanced cardiac life support; CPR:* cardiopulmonary resuscitation*; OHCA:* out-of-hospital cardiac arrest; *“Signs of life”:* definite directed movements (neither simple breathing/gasping efforts nor undirected moving)

**Table S2:** Cannulation-related complications and ECMO technical issues (n=213)

|  | **N (%)** |
| --- | --- |
| **Acute cannulation-related complications** | 37 (17.4) |
| Malposition  Vessel perforation  Site bleeding  Retroperitoneal bleeding  Cannulation failure | 12 (32.4)  8 (21.6)  7 (18.9)  6 (16.2)  4 (10.8) |
| **Technical ECMO problems** | 18 (8.5) |
| Oxygenator-related  Pumphead-related  Pump console-related  Circuit-related  Others | 7 (38.9)  2 (11.1)  2 (11.1)  4 (22.2)  3 (16.7) |

*Notes: ECMO: extracorporeal membrane oxygenation*

**Table S3:** Cannulation-related complications over a 10-year period (n=213)

|  | **October 2013 – December 2020** | **January 2021 – September 2023** |
| --- | --- | --- |
| **Complication rate** including bleeding on cannulation side, malposition of cannula, vessel perforation | 21,9%  (30/137) | 9,2%  (7/76) |

**Table S4:** Cause of mortality in ECPR patients (n=139)

| **Reasons of mortality** | **N (%)** |
| --- | --- |
| Cerebral hypoxia | 98 (70.5) |
| Refractory low cardiac output  (with contraindication for LVAD/BIVAD) | 15 (10.8) |
| Multi-organ failure | 10 (7.2) |
| Bleeding (not intracranial) | 8 (5.7) |
| Cerebral bleeding | 4 (2.9) |
| Intestinal ischemia | 3 (2.2) |
| Sepsis | 1 (0.7) |
